# Shenlian Extract Against Myocardial Injury Induced by Ischemia Through the Regulation of NF-κB/IκB Signaling Axis

**DOI:** 10.3389/fphar.2020.00134

**Published:** 2020-03-06

**Authors:** Yuan Guo, Qing Yang, Xiao-Gang Weng, Ya-Jie Wang, Xue-Qi Hu, Xiao-Jun Zheng, Yu-Jie Li, Xiao-Xin Zhu

**Affiliations:** ^1^Pharmacokinetics Laboratory, Institute of Chinese Materia Medica, China Academy of Chinese Medical Sciences, Beijing, China; ^2^College of Pharmacy, Tianjin University of Traditional Chinese Medicine, Tianjin, China; ^3^Pharmacy Department, First Hospital of Shanxi Medical University, Taiyuan, China

**Keywords:** Shenlian extract (SL), ischemic heart disease (IHD), myocardial ischemic injury, NF-κB signaling pathway, inflammation

## Abstract

Ischemic heart disease (IHD), caused predominantly by atherosclerosis, is a leading cause of global mortality. Our previous studies showed that Shenlian extract (SL) could prevent the formation of atherosclerosis and enhance the stability of atherosclerotic plaques. To further investigate the protective effects of SL on myocardial ischemic injury and its possible mechanisms, anesthetized dogs, *ex vivo* rat hearts, and H9c2 cardiomyocytes were used as models. The results showed that SL had a significant protective effect on the anesthetized dog ligating coronary artery model, reduced the degree of myocardial ischemia (Σ-ST), and reduced the scope of myocardial ischemia (N-ST). Meanwhile, SL alleviated ischemic reperfusion damage in *ex vivo* rat hearts with improved LVEDP and ± dp/dt_max_ values of the left ventricle. SL reduced the pathological changes of LDH, IL-1β, MDA, and NO contents, all of which are related to the expression of NF-κB. Further analysis by Bio-Plex array and signal pathway blocker revealed that the phosphorylation of IκB was a key factor for SL to inhibit myocardial ischemic injury, and the regulation of SL on IκB was primarily related to degradation of the IκB protein. These results provided dependable evidence that SL could protect against myocardial ischemic injury through the NF-κB signaling pathway.

## Introduction

Cardiovascular disease (CVD) remains the leading cause of global mortality. Ischemic heart disease (IHD), is the second leading cause of death for the Chinese population (Zhao et al., 2019), is one of the main clinical manifestations of CVD (Herrington et al., 2016). It has been reported that mortality caused by IHD is common in Eastern Europe and Asia (Moran et al., 2014; Akbalaeva et al., 2017). Myocardial ischemic disease has risen to a top cause of morbidity and mortality worldwide (Zhao et al., 2019). The development of atherosclerosis in the coronary artery is the underlying pathological mechanism of myocardial ischemia and myocardial infarction (Foks and Bot, 2017). In the most serious situations, an advanced atherosclerotic plaque can rupture or erode, leading to the formation of an occluding thrombus and the subsequent incidence of an acute cardiovascular event. Simultaneously, inflammation, which is an emerging risk factor, leads to the accumulation of lipids and inflammatory cells in the vascular wall (Bertrand and Tardif, 2017). Many experimental and clinical studies support interleukin-1 beta (IL-1β) secretion, which targets vascular cells, as a promising research target to find new anti-atherosclerotic agents that better target inflammation (Hansson, 2017; Libby, 2017). Interventions targeting reductions in inflammation to prevent plaque instability are therefore required. Along with contemporary interventions and pharmacologic agents, traditional Chinese medicine (TCM) has been extensively prescribed in clinics in China and some Asian countries (Wang et al., 2017). TCM has attracted increasing attention around the world, because the treatment consists of significant bioactivity by multiple components (Xu et al., 2015; Zhang et al., 2015).

Shenlian extract (SL) is a combination of *Salvia miltiorrhiza Bunge* and *Andrographis paniculata* extract. Based on the clinical experience of TCM, SL can prevent and treat atherosclerosis and cardiovascular diseases (Zhou et al., 2013; Guo et al., 2016). The mechanism of action of *S. miltiorrhiza Bunge* includes promoting the circulation of blood, removing blood stasis, improving blood rheology by inhibiting platelet aggregation, and changing erythrocyte deformability or reducing plasma viscosity (Tang et al., 2002; Li et al., 2004). Via these mechanisms, it is shown to inhibit myocardial apoptosis after myocardial ischemic reperfusion injury in rats (Song et al., 2013). Furthermore, modern pharmacological studies have shown that *S. miltiorrhiza Bunge* also affects myocardial remodelling to improve myocardial infarction disease (Wang et al., 2018). The other ingredient, *A. paniculata*, has an anti-inflammation and detoxification effect (Pang et al., 2016; Zou et al., 2016), improving the hemodynamic and ventricular function against ischemic reperfusion injury (Ojha et al., 2012) and protecting endothelial cells (You et al., 2011).

Previous studies have demonstrated that SL has a protective effect on atherosclerosis plaque formation, significantly reducing the plaque area of atherosclerosis, reducing the severity of the pathological condition (Li et al., 2011), lowering the blood lipid levels by improving lipid metabolism, and reducing the infiltration and deposition of lipids in the blood vessel wall (Guo et al., 2016). Furthermore, SL has a protective effect by inhibiting the infiltration and adhesion of local inflammatory cells, improving the local blood flow of vessels, and inhibiting the cytoskeletal rearrangement after injury of vascular endothelium. Moreover, SL stabilized atherosclerosis plaques. Through an ApoE^–/–^ mouse model with vulnerable plaques, which were induced by combined multi-factor induction, SL was reported to reduce the levels of macrophagocytes and lipids in plaque, decrease the expression levels of MMP-2 and MMP-9 in plaques, and up-regulate the contents of collagen fibers and expression of plaques (Guo et al., 2016). In addition, SL decreased the proliferation and degranulation of mast cells in extravascular mucosa induced by neuropeptide P, and reduced the inflammatory response and intraplaque hemorrhage of the plaque (Li Y. J. et al., 2016).

We propose that SL could synthetically affect the formation process of atherosclerosis through various pathways mainly inflammation responses. A primary goal of this report is to further explore the protective effect of SL on myocardial ischemic injury and its possible mechanism based on NF-κB signaling pathway.

## Materials and Methods

### Materials and Reagents

Nitro blue tetrazolium chloride (N-BT) was purchased from the medicine supply station of the Academy of Military Medical Sciences (China). Lactate dehydrogenase (LDH), malondialdehyde (MDA), nitric oxide (NO), nitric oxide synthase (NOS), IL-1β, PI3K, and nuclear factor kB (NF-kB) kits were purchased from Nanjing Jiancheng Bioengineering Institute (China). High glucose Dulbecco's modified Eagle's medium (DMEM-H) and fetal bovine serum (FBS) were purchased from Gibco. MicroRotofor™ cell lysis kit and Bio-Plex Pro™ cell signaling MAPK panel were purchased from Bio-Rad (USA). Proteasome inhibitor, MG132, and IKK (inhibitor of nuclear factor kappa-B kinase, IκB) inhibitor, parthenolide, were purchased from Sigma.

### Plant Materials

SL consisted of *S. miltiorrhiza Bunge* extract and *A. paniculata* extract at a ratio of 15:9. *S. miltiorrhiza Bunge* and *A. paniculata* originated from the Linyi, Shandong province and Guangxi province (China). The taxonomic authenticity was identified by Prof. Xirong He of the Institute of Chinese Materia Medica of the China Academy of Chinese Medical Sciences (Beijing, China). *S. miltiorrhiza Bunge* was identified as the dry rhizome of *S. miltiorrhiza Bge.*, and *A. paniculata* was identified as the herba of *A. paniculata* (Burm F.) Nees. The *S. miltiorrhiza Bunge* extract included two types of components. One component was extracted with ethanol under percolation and then concentrated under reduced pressure, and the other component was prepared by dilute ethanol soaking and purified by microporous resins SP825. The *A. paniculata* extract was prepared by dilute ethanol soaking, and purified by macroporous resins SP825. The extract of SL primarily contained tanshinone IIA (3%), salvianolic acid B (38%) and andrographolide (20%). The extraction rate of the water-soluble partial extract of *S. miltiorrhiza Bunge* was 2.27%, and that of the fat-soluble partial extract was 1.31%. The extraction rate of the partial extract of *A. paniculata* was 2.11%. (Guo et al., 2016).

### High Performance Liquid Chromatography (HPLC) Analysis of SL

SL extract was detected using Waters HPLC. The conditions of the HPLC are shown in **Table 1**, and the HPLC chromatogram of SL can be found in the **Supplementary Material**.

**Table 1:** High performance liquid chromatography (HPLC) conditions of Shenlian extract (SL).

	Andrographolide	Tanshinone IIA	Salvianolic acid B
Instrument		Waters HPLC	
Detector		2489 UV detector	
RP column	Phenomenex Synerigi Hydro-RP C18 (250×4.6mm, 4µm)	Phenomenex Gemini C18 (250×4.6mm, 5µm)	Phenomenex Gemini C18 (250×4.6mm, 5µm)
Column temperature		Indoor temperature	
Injection vol		10 μL	
UV wavelength	286 nm	228 nm	270 nm
Mobile phase	Methanol-Acetonitrile-Water (30/10/60, 0.1vol.% anhydrous formic acid)	Methanol-Water (45/55, v/v)	Methanol-Water (75/25, v/v)

### Experimental Animals and Grouping

Healthy Beagle dogs (11.2 ± 2.5 kg), both male and female, were obtained from Beijing Shahe Tongli Experimental Animal Farm. The animal studies were approved in accordance with the recommendations in the Guidance for the Care and Use of Laboratory Animals issued by the Ministry of Science and Technology of China and the Use Committee of Institute of Basic Theory for Chinese Medicine, China Academy of Chinese Medicine Science. These dogs were randomly assigned into four groups (six dogs in each group): myocardial ischemia model group (MI, operated with ligating left anterior descending), positive control diltiazem treatment group (treated with at 5 mg/kg diltiazem), low dosage SL treatment group (66 mg/kg), and high dosage SL treatment group (132 mg/kg).

The adult male Wistar rats (250-300g) were obtained from Beijing Vital River Laboratory Animal Technology Co., Ltd. The animal studies were also performed in accordance with the Guidance for the Care and Use of Laboratory Animals issued by the Ministry of Science and Technology of China and the Use Committee of Institute of Basic Theory for Chinese Medicine, China Academy of Chinese Medicine Science. The rats were randomly assigned into the following five experimental groups (eight rats in each group): vehicle control group (control, operated with continuous perfusion); ischemia reperfusion control group (I/R, operated with at ischemia and reperfusion), positive control verapamil treatment group (150 ng/kg), low dosage SL treatment group (224 mg/kg), and high dosage SL treatment group (448 mg/kg).

All experimental procedures and animal management were approved and performed by Institute of Chinese Materia China Academy of Chinese Medical Science. All animals were maintained under controlled conditions of temperature (24 ± 2°C) and humidity (55 ± 5%), a 12-hour light/dark cycle, and allowed free access to food and water.

### Procedure of Myocardial Ischemic Model

Dogs were maintained with free access to regular diet and distilled water for one week before surgery. The model of myocardial ischemia in anesthetized dogs was established as previously described (Berger et al., 1976; Fu et al., 2013). Briefly, Beagle dogs were anesthetized with 1% pentobarbital sodium 30 mg/kg by intravenous injection. After endotracheal intubation, the chest of the dog was opened by left thoracotomy to expose the heart, and the left anterior descending (LAD) coronary artery was ligated. Finally, 15 min after ligation, drugs were given by duodenal injection. Inclusion criteria for the study were that the ST segment increased more than 2 mV. After ligation, the multi-point epicardial electrode (30 landmarks) was sutured on the ventricular surface, and the epicardial electrocardiogram (EECG) was connected by the multi-channel physiological instrument (BIOPAC, USA). Myocardial ischemia degree (total mV of ST-segment elevation, Σ-ST) and the myocardial ischemia scope (total number of ST-segment elevation points, N-ST) were counted.

### Measurement of Myocardial Infarction Area in Dogs by N-BT Staining

To detect myocardial infarction size, which is closely related to the left ventricular ejection fraction and cardiac function, N-BT staining was used. The N-BT non-stained area represents the infarcted area, and the N-BT stained area represents the non-infarcted area. The heart was removed and divided into five pieces. Next, the heart was cut parallel to the coronary sulcus and placed into the N-BT staining solution for 15 min at room temperature after EECG testing last 180 min. The medical image analysis system (HPIAS-1000, Beijing, China) was used to measure the infarcted area (N-BT non-stained area) and non-infarcted area (N-BT stained area) on each side. The percentage of infarcted area of the ventricle and total heart was calculated.

### Procedure of Langendorff-Perfused Rat Hearts Model

**Figure 1:**
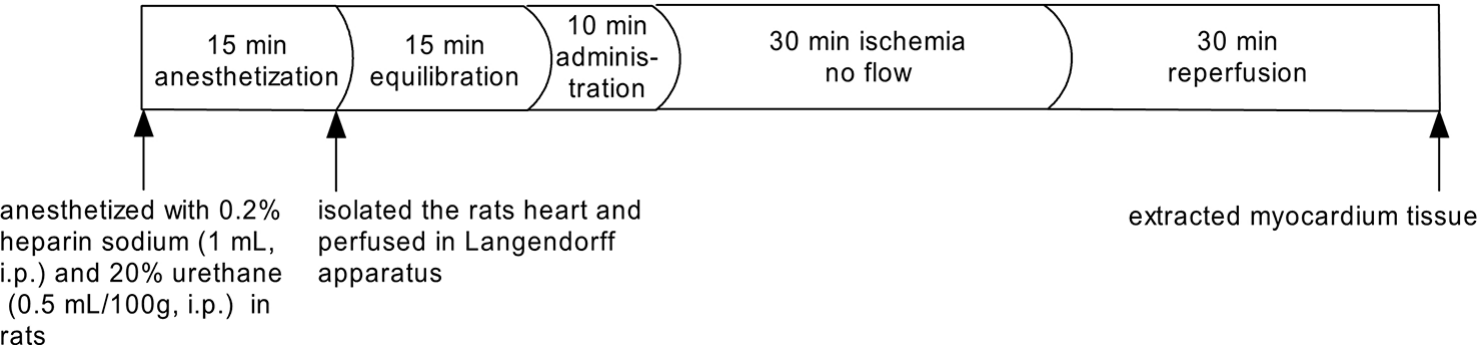
Experimental protocol of ischemia–reperfusion *via* Langendorff.

Rats were anesthetized with 0.2% heparin sodium (1 mL, i.p.) and 20% urethane (0.5 mL/100g, i.p.) after 10 min. The hearts were quickly removed, and the aorta was cannulated and retrogradely perfused at constantly pressure (70–80 mmHg) on a Langendorff apparatus (LE3/42502002, Panlab, Spain) with a nutrient rich oxygenated KH (Krebs-Henseleit) solution maintained at 37 ± 1°C with a physiological pH of 7.4 (Ytrehus, 2000; Bell et al., 2011). A self-made latex balloon was inserted in the left ventricle of the heart, and a pressure transducer was placed on the other side of the balloon (DA100C, BIOPAC, USA). The experimental procedure of perfused the heart is summarized in **Figure 1**. The following parameters of myocardial function were measured during equilibration time and at 5, 10, 15, 20, 25, and 30 min during reperfusion by the MP150 multi-channel physiological instrument (BIOPAC, USA): the pressure curve of the left ventricle, the left ventricular systolic pressure (LVSP), the left ventricular end diastolic pressure (LVEDP), and the left ventricular internal pressure maximal rise and maximum decline rate (± dp/dt_max_).

### Determination Biochemical Parameters of Myocardium Tissue of Rats

The myocardium tissue obtained from the re-perfused hearts were used for evaluating the concentration of LDH, MDA, NO, and NOS, which are routine indicators commonly used for diagnosing myocardial inflammation and acute myocardial infarction. After reperfusion for 30 min, the heart was immediately removed and contained approximately 0.4 g of apical tissue. Then, 10% (w/v) of fresh myocardial homogenate was prepared and centrifuged at 3,000 rpm for 15 min. The supernatant was collected to assay the levels of biochemical parameters using chemical colorimetry of kits, and specific operations was strictly followed by the manufacturer’s instructions.

### Measurement of Inflammatory Parameters by ELISA

The PI3K pathway plays an important role in regulating a variety of cellular functions including survival, transcription and protein synthesis. NF-κB is activated downstream of PI3K.The supernatant of fresh myocardial homogenate was acquired to measure the concentrations of IL-1β, PI3K and NF-κB by commercial enzyme linked immunosorbent assay (ELISA) kits, and the detection operations were performed according to the manufacturer’s instructions.

### H9c2 Cell Culture

H9c2 rat embryonic cardiomyocytes (from National Infrastructure of Cell Line Resource) were cultured in DMEM-H supplemented with 10% FBS. All cells were cultivated at 37°C in a humidified incubator maintained at 5% CO_2_. Cells grown to sub-confluence were used to complete all the based cell experiments.

### Measurement of H_2_O_2_-Induced Oxidative Cell Damage in H9c2 Cells

SL was mixed with the DMEM-H medium to make a stock solution with a concentration of 1 mg/mL. The dimethyl sulfoxide (DMSO) concentration in the stock solution was 0.1% v/v, and then the solution was stored in –20°C. The cells were treated with SL at a final concentration of 10, 25, or 50 μg/mL, diluted with with DMEM-H medium. The SL treatment groups were pre-treated with the corresponding drugs for 24 h before H_2_O_2_ induction, and the cells were exposed to H_2_O_2_ (500 μM) for 6 h to induce cells damage. The cells were divided into five groups: a untreated control group (untreated); a H_2_O_2_-induced group (500 μM H_2_O_2_ for 6 h); a 50 μg/mL SL treatment group (SL 50 μg/mL treatment for 24 h, then 500 μM H_2_O_2_ for 6 h); a 25 μg/mL SL treated group (SL 25 μg/mL treatment for 24 h, then 500 μM H_2_O_2_ for 6 h); a 10 μg/mL SL treated group (SL 10 μg/mL treatment for 24 h, then 500 μM H_2_O_2_ for 6 h). H9c2 cells seeded at 5 × 10^4^ cells in 24 wells in DMEM-H and 10% FBS. The cells were cultured overnight, and then treated with SL extracts for 24 h and H_2_O_2_ (500 μM) for 6 h. The supernatant of the cell culture was collected. The concentrations of LDH and NO were determined by chemical colorimetry kits, and the concentrations of IL-1β was determined by ELISA. The instructions from the kit were strictly followed.

### Bio-Plex Array Analysis in H9c2 Cells

H9c2 cells were lysed by MicroRotofor™ Cell Lysis Kit, and centrifuged at 15,000×g for 10 min at 4°C. The supernatant was collected, and its protein concentration was detected by the Bradford method. Following the kit’s instruction, the Bio-Plex Pro™ Cell Signaling MAPK Panel was used to detect the following phosphorylated analytes from cell lysates: p-IκB-α, p-ERK1/2, p-p38 MAPK, p-Stat3, p-c-JUN, p-IGF-IR, p-MEK, p-Akt. The Bio-Plex cytokine assay buffer was used, the Bio-Plex array reader collects the data that were analyzed by the Bio-Plex^®^ 200 Systems (Bio-Rad, USA).

### Immunofluorescence Analysis in H9c2 Cells

In the SL treatment groups, the proteasome inhibitor, MG132 (5 μM), and IKK inhibitor, parthenolide (10 μM), were incubated in culture cells for 12 h before drug treatment to explore whether degradation or phosphorylation, respectively, modulated the IκB protein (Bergmann et al., 2001; Planavila et al., 2005). The function pathway was examined by comparing the expression of filamentous actin (F-actin) in the groups of H9c2 cells. Staining with fluorescently labeled phalloidin clearly showed the distribution of microfilaments in the cells (Lukinavičius et al., 2014). The expression of F-actin in the cells were observed by Laser Scanning Confocal Microscope (Olympus, Janpan) immediately after labelling, with excitation wavelength of 488 nm and emission wavelength of 530 nm, per instructions. The supernatant of cells was collected to detect the levels of NO by chemical colorimetry and IL-1β by ELISA to confirm the oxidative stress and inflammation levels in injured cells, respectively.

### Statistical Analysis

All experimental data were analyzed by GraphPad Prism 5.0 and plotted as the mean ± standard error of the mean (SEM). Results were performed through one-way analysis of variance (ANOVA) and Dunnett's Multiple Comparison test to determine statistical significance; P-values less than 0.05 were defined as statistically significance.

## Results

### SL Reduced the Degree and Scope of Myocardial Ischemia on Anesthetized Dogs

**Figure 2:**
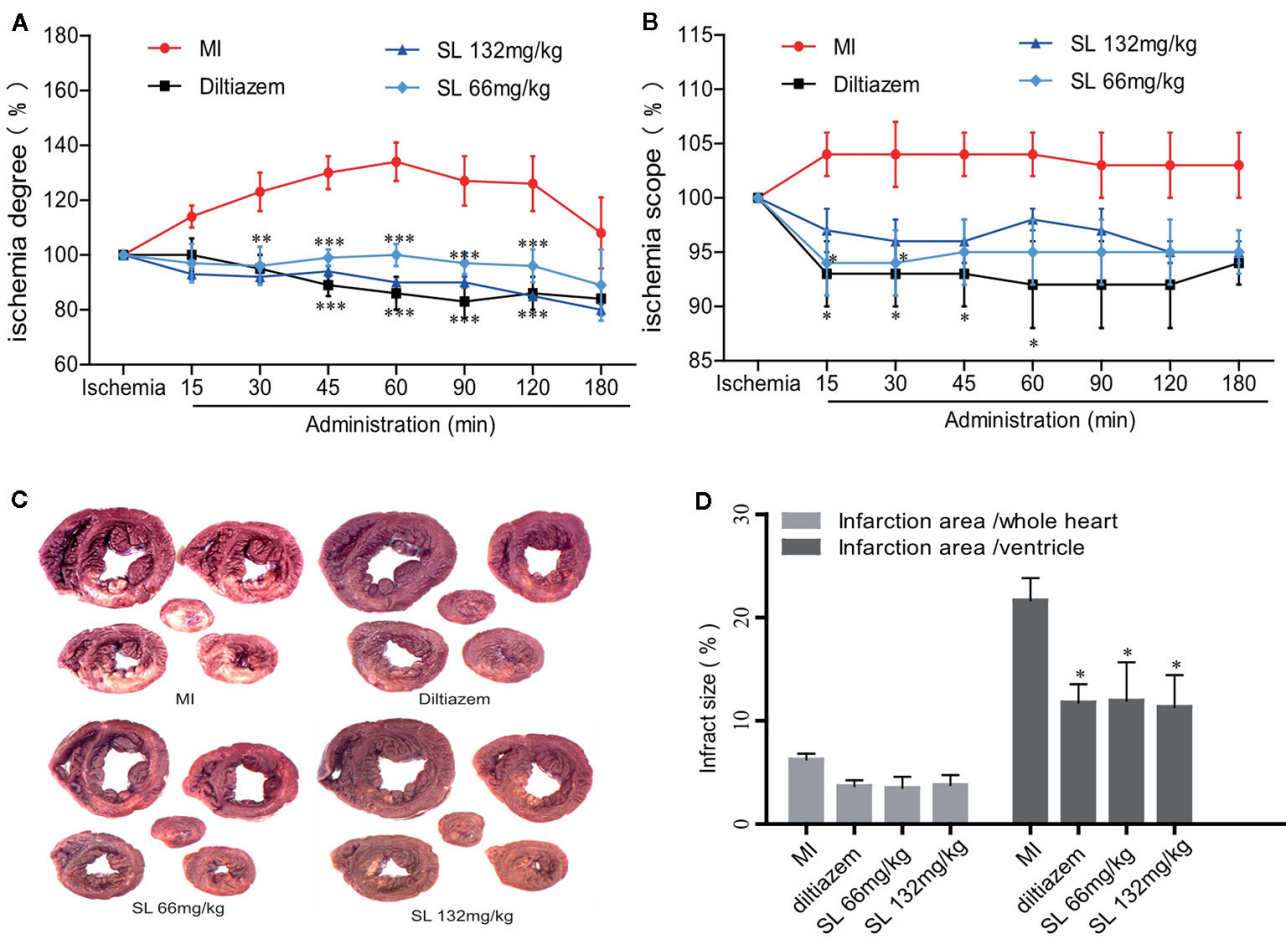
Effects of Shenlian extract (SL) on myocardial ischemia in anesthetized dogs. **(A)** Effects of SL on the degree (Σ-ST) of myocardial ischemia indicated by epicardial electrocardiogram (EECG). **(B)** Effects of SL on the scope (N-ST) of myocardial ischemia as indicated by EECG. **(C)** Effects of SL on the area of myocardial infarction, represented by N-BT staining of the myocardial infarct. **(D)** Effects of SL on the infarction area, quantification of infarction area. Results represent mean ± SEM, n = 6. Statistical analysis was performed by one-way ANOVA, followed by Dunnett's Multiple Comparison test. *P < 0.05, *** *P* < 0.001 versus myocardial ischemia (MI) group.

An EECG was performed to determine the real-time effect of SL on myocardial ischemic dog hearts. After ligation of LAD in the MI group, the ischemia degree (Σ-ST) increased markedly and continued to increase during the entire process (**Figure 2A**). Compared to the MI group, the Σ-ST significantly decreased after 66 mg/kg of SL (*P* < 0.05). At 180 min after administration, the ischemia degree of dogs treated with 66 mg/kg and 132 mg/kg of SL was reduced by 89% and 80%, respectively. Similarly, the ischemia scope (N-ST) significantly decreased after the administration with 66 mg/kg of SL at 15 and 30 min (*P* < 0.05, **Figure 2B**).

### SL Reduced the Area of Myocardial Ischemia on Anesthetized Dogs

The myocardial infarction size was estimated by quantitative histological N-BT staining. Visually, the infarction size of the SL treatment groups was smaller than that of the MI group (**Figure 2C**). Compared to the MI group, the percentage of ventricular infract size in the SL 132mg/kg group was 11.4 ± 5.1%, which was significantly reduced (*P* < 0.05, **Figure 2D**), and that in the SL 66mg/kg group was significantly reduced to 12.0 ± 9.1% (*P* < 0.05). In contrast, the whole heart infarct size was 3.8 ± 1.8% in the SL 132mg/kg group and 3.5 ± 2.7% in the SL 66mg/kg group. Overall, these data suggest that SL treatment attenuated myocardial injury in anesthetized dogs.

### SL Alleviated Myocardial Ischemia–Reperfusion Injury in Isolated Rat Hearts

**Figure 3:**
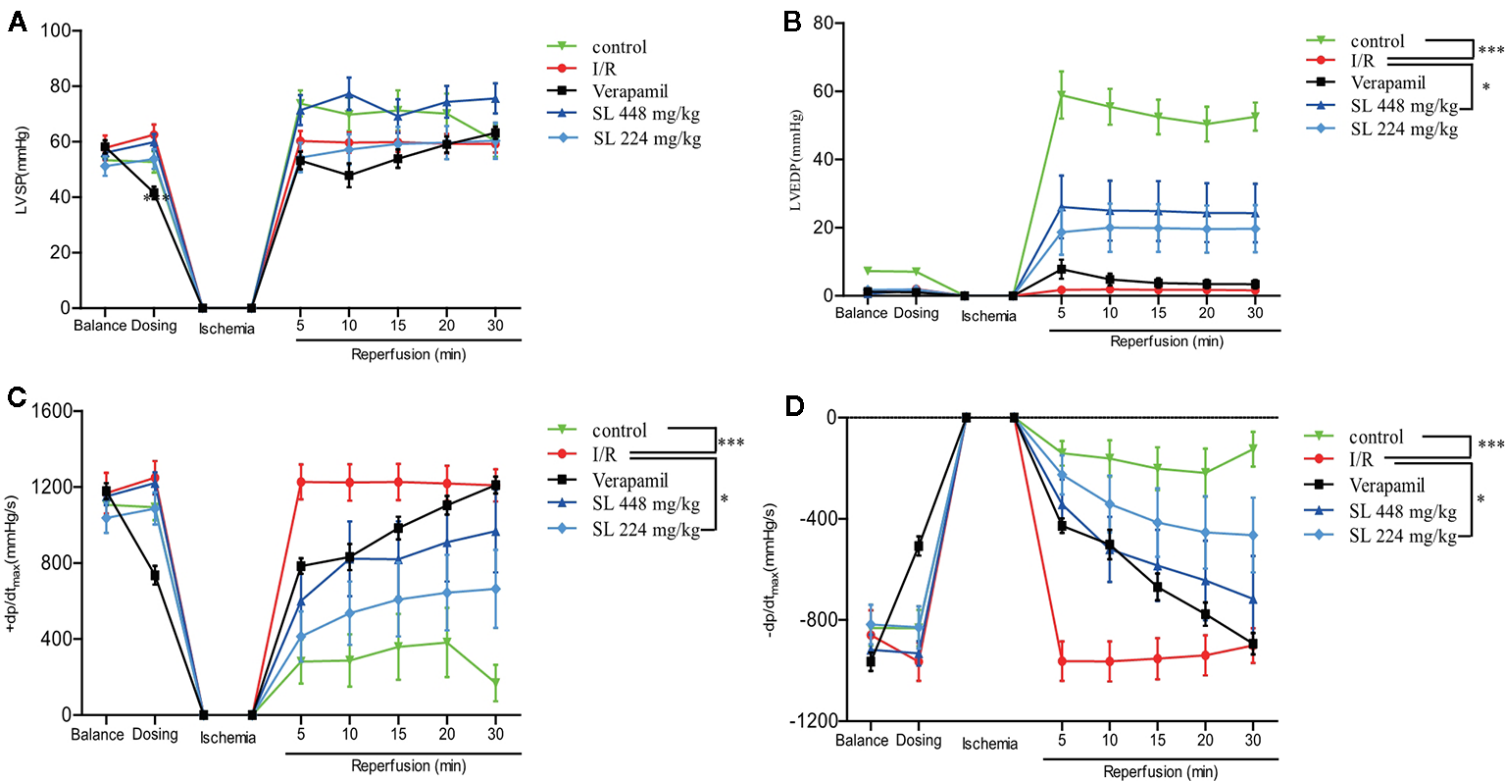
Effects of Shenlian extract (SL) on left ventricular function in isolated rat hearts. **(A)** Changes in the left ventricular systolic pressure (LVSP) by myocardial ischemia and reperfusion by MP150 detection. **(B)** Changes in left ventricular end diastolic pressure (LVEDP) by myocardial ischemia and reperfusion by MP150 detection. **(C)** Changes in + dp/dt_max_ by myocardial ischemia and reperfusion calculated by left ventricular pressure curves. **(D)** Changes in -dp/dt_max_ by myocardial ischemia and reperfusion calculated by left ventricular pressure curves. Results represent mean ± SEM, n = 8. Statistical analysis was performed by one-way ANOVA, followed by Dunnett's Multiple Comparison test. * *P* < 0.05, *** *P* < 0.001 versus the ischemia reperfusion (I/R) group.

A self-made latex balloon connected to a pressure transducer was inserted into the left ventricle. The left ventricular pressure curve was recorded to evaluate heart function in the dogs. During the process of ischemia–reperfusion injury, KH solution was injected into the balloon to make the LVEDP between 0-10 mmHg. The normal value of the LVSP was 50-60 mmHg when the heart was perfused and balanced for 15 min, but LVSP had no significant change of the SL treatment groups compared with the I/R group (**Figure 3A**). After 30 min of ischemia, 448 mg/kg of SL induced a significant increase in LVEDP compared with the I/R group (*P* < 0.05, **Figure 3B**). The myocardial diastolic function was reflected by ± dp/dt_max_ during the isovolumic contraction phase. The data suggest that there was a significant decreased in both in + dp/dt_max_ and – dp/dt_max_ in the SL 224 mg/kg group compared with the I/R group (*P* < 0.05, **Figures 3C, D**).

### SL Protected the Myocardium From Ischemia–Reperfusion Injury by Elevating The Concentration of NF-κB in Isolated Rat Heart

**Figure 4:**
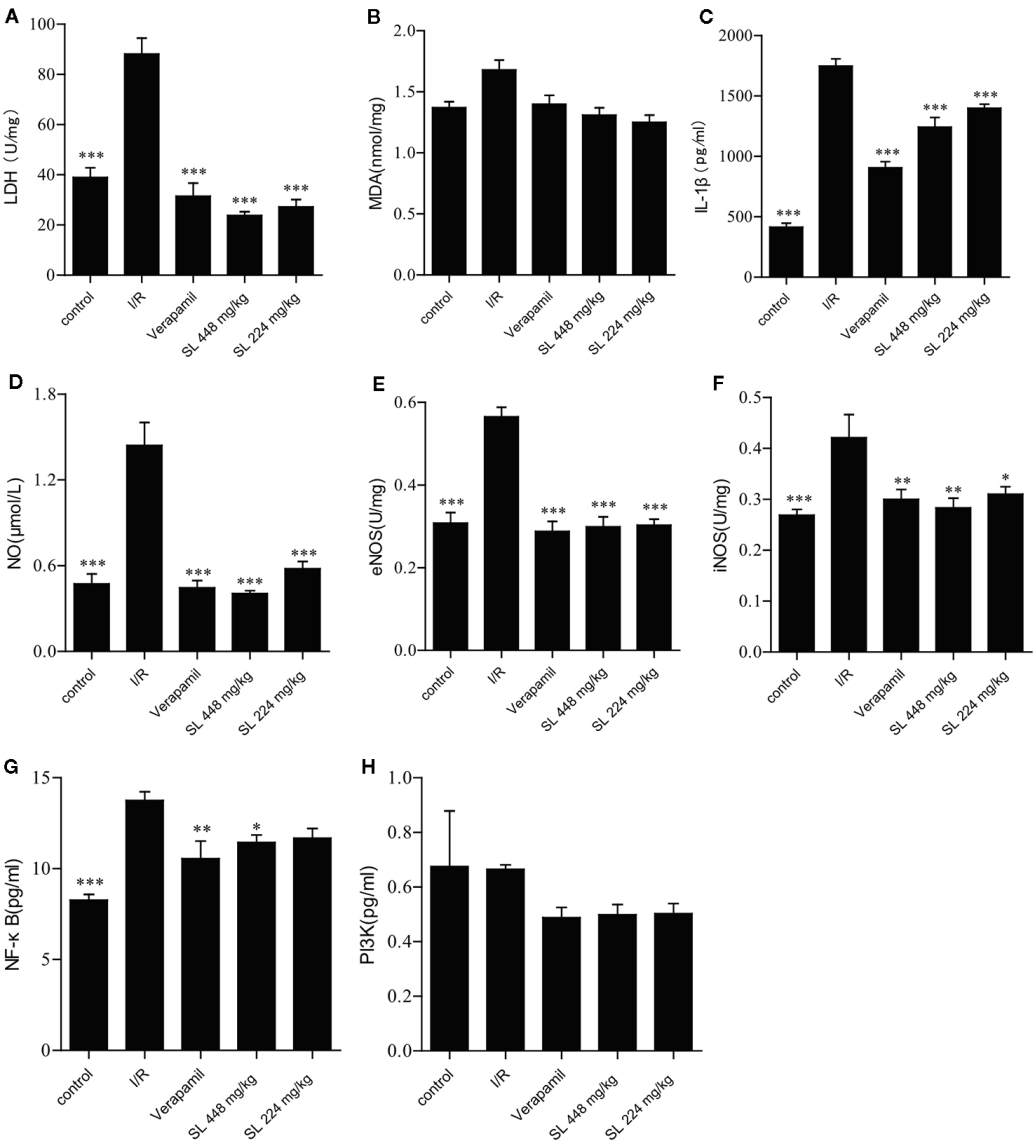
Effects of Shenlian extract (SL) on biochemical parameters in isolated rat hearts. **(A)** Effect of SL on lactate dehydrogenase (LDH) contents in isolated rat hearts. **(B)** Effect of SL on malondialdehyde (MDA) contents in isolated rat hearts. **(C)** Effect of SL on interleukin (IL)-1β contents in isolated rat hearts. **(D)** Effect of SL on nitric oxide (NO) contents in isolated rat hearts. **(E)** Effect of SL on nitric oxide synthase e(NOS) contents in isolated rat hearts. **(F)** Effect of SL on iNOS contents in isolated rat hearts. **(G)** Effect of SL on NF-κB contents in isolated rat hearts. **(H)** Effect of SL on PI3K contents in isolated rat hearts. Results represent mean ± SEM, n = 8. Statistical analysis was performed by one-way ANOVA, followed by Dunnett's Multiple Comparison test. * *P* < 0.05, ** *P* < 0.01, *** *P* < 0.001 versus the ischemia reperfusion (I/R) group.

After ischemia and 30 min of reperfusion, myocardial homogenate was made from the heart to detect the biochemical parameters. The levels of LDH, IL-1β and NO were markedly increased (*P* < 0.001, **Figures 4A, C, D**), indicating obvious myocardial injury after the myocardial ischemia–reperfusion. After SL administration, LDH, IL-1β and NO were markedly decreased compared with the I/R group (*P* < 0.001). No differences in MDA concentration between the groups (**Figure 4B**). Collectively, the above-mentioned results suggest that SL treatment protected the isolated heart.

NO regulates blood pressure and maintains blood flow distribution in tissues and organs through vasodilatation. Additionally, NO directly participates in the inflammatory response, and inhibits platelet aggregation and adherence of neutrophils. Some studies have found that the metabolites of NO have strong cytotoxicity and damage cells (Yamaoka et al., 2002). Our results displayed a marked decrease in NO (*P* < 0.001) compared with I/R after SL treatment (**Figure 4D**), evidencing effective control of the typical inflammatory response. Endothelial nitric oxide synthase (eNOS), as an isozyme of NOS, is one of key factors that regulates vascular function. Through production of. NO causes angiectasis and superoxide, which causes vasoconstriction (Lisakovska et al., 2017). Simultaneously, inducible NO synthase (iNOS), another isozyme of NO, is the primary rate-limiting enzyme of the inflammatory response. iNOS and eNOS were significantly decreased in the SL groups compared with the I/R group (*P* < 0.05, **Figures 4E, F****)**, indicating that SL protected the heart by regulated vascular function.

The PI3K/Akt signaling pathway regulates cardiac function through endothelial cells migration, angiogenesis, ventricular remodeling, and other mechanisms. Akt is phosphorylated after abnormal activation of PI3K pathway, which in turn activates the expression of NF-κB. As NF-κB is a key factor in regulation of the inflammatory signaling networks, the activation of NF-κB not only up-regulates the expression of many pro-inflammatory genes encoding inflammatory mediators and cytokines, including IL-1β, IL-1, and TNF-α, but also activates related enzymes, such as iNOS and vascular cells adhesion molecules, regulating the production of NO, and amplifying the cascade of the inflammatory responses. Our study showed that NF-κB expression was significantly decreased (*P* < 0.05) in the SL 448 mg/kg group compared with the I/R group (**Figure 4G**), but there was no change in the concentration of PI3K between groups (**Figure 4H**). Collectively, SL had a protective effect against NF-κB, similar to the effect against IL-1β. Therefore, SL likely protected against ischemia–reperfusion injury through NF-κB pathway.

### SL Protected H9c2 Cells From H_2_O_2_-Induced Damage

**Figure 5:**
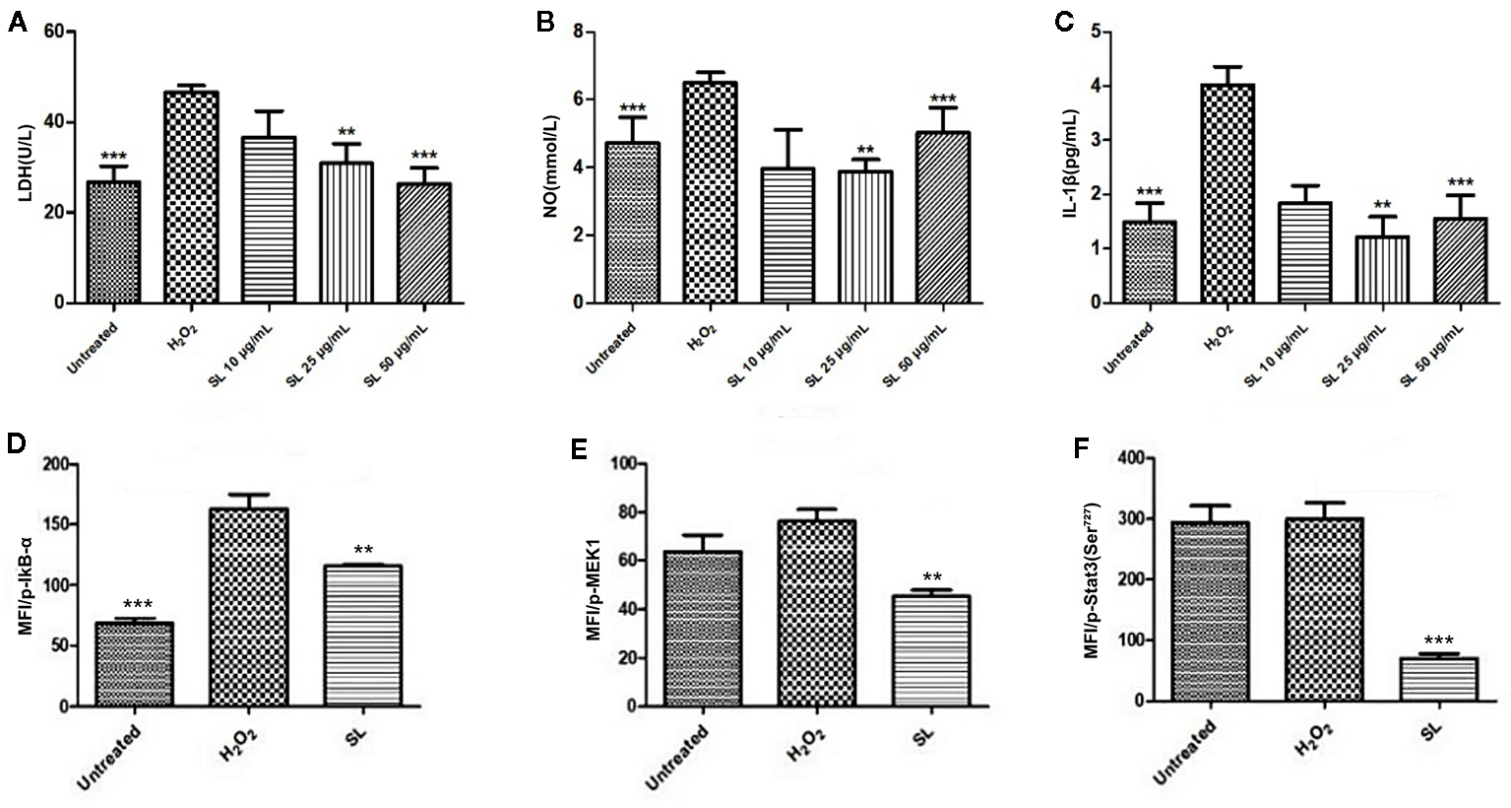
Effect of Shenlian extract (SL) on H_2_O_2_-induced damage in H9c2 cells. **(A)** Effect of SL on lactate dehydrogenase (LDH) contents in H9c2 cells by H_2_O_2_-induction for 6 h. **(B)** Effect of SL on nitric oxide (NO) contents in H9c2 cells by H_2_O_2_-induction for 6 h. **(C)** Effect of SL on interleukin (IL)-1β contents in H9c2 cells by H_2_O_2_-induction for 6 h. **(D)** Effect of SL on p-IκB in H9c2 cells by H_2_O_2_-inductionfor 6 h by Bio-Plex array analysis. **(E)** Effect of SL on p-MEK1 in H9c2 cells by H_2_O_2_-induction for 6 h by Bio-Plex array analysis. **(F)** Effect of SL on p-Stat3 in H9c2 cells by H_2_O_2_-induced for 6 h by Bio-Plex array analysis. Results represent mean ± SEM of three independent experiments. Statistical analysis was performed by one-way ANOVA, followed by Dunnett's Multiple Comparison test. **P < 0.01, ***P < 0.001 the H_2_O_2_-induced (H2O2) group.

Based on the results in anesthetized dogs and isolated rat hearts, H9c2 cells induced by H_2_O_2_ were used to identify key targets responsible for the effect of SL. After H_2_O_2_-induction, LDH concentration increased significantly compared with the untreated group (*P* < 0.001, **Figure 5A**). After SL treatment, LDH significantly decreased in the SL 50 μg/mL (*P* < 0.001) and 25 μg/mL (*P* < 0.01) groups compared with the H_2_O_2_ group. Similar to the LDH contents, NO and IL-1β expression decreased significantly in the SL 50 μg/mL (*P* < 0.001) and 25 μg/mL (*P* < 0.01) groups compared with the H_2_O_2_ group (**Figures 5B, C**). These results revealed that SL could reduce the concentration of cellular damaged indicators, and effectively protect the cells from injury and inflammatory response.

### NF-κB Was One of the Important Pathways for the Protective Effect of SL Against Myocardial Ischemic Injury

The phosphorylation levels of eight related signal transduction proteins were detected to explore the mechanism of SL treatment. The phosphorylation levels of IκB-α in H_2_O_2_ group were significantly increased compared with the untreated group, and 25 μg/mL of SL could reduce the expression of p-IκB-α compared with the H_2_O_2_ group (*P* < 0.01, **Figure 5D**). However, there were no significant changes in p-ERK1/2, p-p38 MAPK, p-c-JUN, p-IGF-IR, and p-Akt expression. And the expression of p-Stat3 (*P* < 0.001) and p-MEK (*P* < 0.01) were significantly decreased after 25 μg/mL of SL compared with H_2_O_2_ group (**Figures 5E, F**).

**Figure 6:**
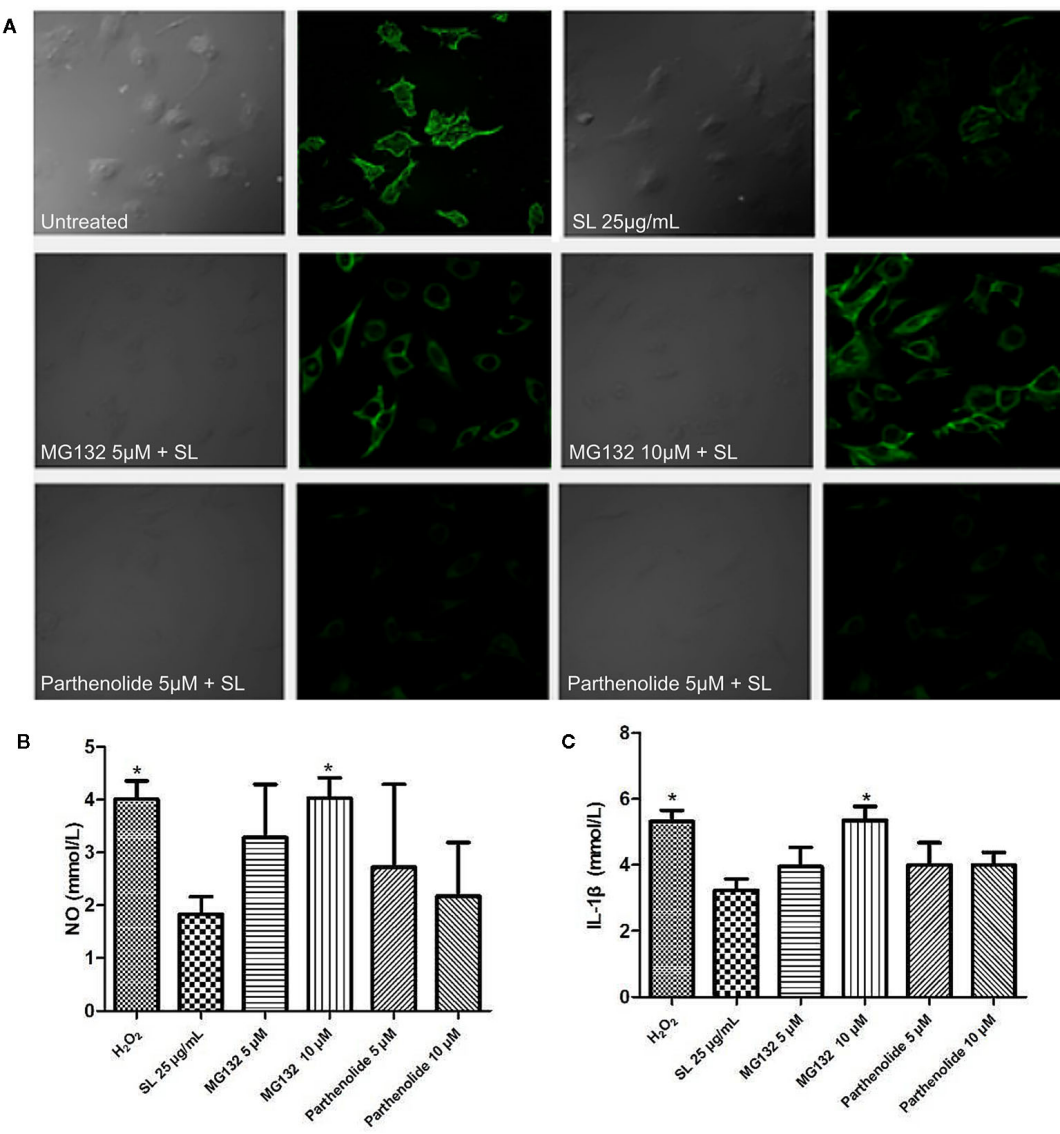
Shenlian extract (SL) affects the degradation of IκB by the proteasome in the NF-κB pathway. **(A)** The expression of F-actin after the proteasome inhibitor, MG132 (5μM, 10μM), and IKK inhibitor, parthenolide (5μM, 10μM), were added to cultivate cells for 12 h before drug treatment. Staining with fluorescently labeled phalloidin, which is displayed in green, clearly shows the distribution of F-actin in the cells. Representative image chosen by 5 visual fields and photos by Laser Scanning Confocal Microscope. **(B)** Effect of SL on nitric oxide (NO) in by H_2_O_2_-induced cells with the added proteasome inhibitor and IKK inhibitor in H9c2 cells for 6 h. **(C)** Effect of SL on interleukin (IL)-1β contents in H_2_O_2_-induced H9c2 cells for 6 h with the added proteasome inhibitor 2 and IKK inhibitor. **(B, C)** represent the mean ± SEM of three independent experiments. Statistical analysis was performed by one-way ANOVA, followed by Dunnett's Multiple Comparison test. * *P* < 0.05 versus the SL 25 μg/mL group.

SL can reduce the expression of p-IκB-α in H_2_O_2_-induced cells *via* IKK kinase activity or protein degradation. Based on the SL-inhibited phosphorylation of IκB, further studies using the proteasome inhibitor, MG132, and IKK inhibitor, parthenolide, were conducted to observe the protective effect of SL in H_2_O_2_ stimulated H9c2 cells. The results suggest that 5 μM of MG132 partially reversed the inhibitory effect of SL on the expression of F-actin in H_2_O_2_-induced cells (**Figure 6A**), and increased NO and IL-1β in the cell supernatant. At a dose of 10 μM of MG132, SL completely reversed the effect of H_2_O_2_ induction, and significantly increased NO and IL-1β contents compared with 25 μg/mL of SL (*P* < 0.05, **Figures 6B, C**). In contrast, 5 μM and 10 μM of parthenolide had no significant effect on SL. These data suggest that the regulation of NF-κB by SL primarily affects the degradation of IκB by proteasome, leading to the inhibition of the nuclear translocation of NF-κB, which affects the activation of NF-κB pathway. This is independent of IKK kinase.

## Discussion

Previous studies have shown that SL inhibits and stabilizes atherosclerosis plaques through inflammatory reactions and plaque destabilization, which inhibits the expression of the inflammatory mediators, IL-10 and MCP-1, further increasing the expression of the metabolite of inflammatory dispersalin, LXA4 (Li et al., 2011). Furthermore, Guo Y (Guo et al., 2016) concluded that SL inhibits the formation of atherosclerotic plaques in the carotid artery and reduces the degree of lesions in ApoE^–/–^ mice. SL regulates lipid metabolism by reducing the inflammatory biomarkers such as TC, TG and LDL. Meanwhile, by established an EC-SMC-MC co-culture system, the study showed that tanshinone IIA, an active ingredient of SL, exhibited significant efficacy against atherosclerosis and inhibited the inflammatory MMP-2 and NF-κB pathway (Li et al., 2015). SL plays a role in protecting against atherosclerosis by intervening against plaque formation and plaque instability.

This is the first report on the direct protection of SL against myocardial ischemic injury. This protective effect was observed, and its mechanism was preliminarily explored. In the animal models, SL treatment in anesthetized dogs and Langendorff-perfused rats had a significant protective effect on myocardial ischemia injury. Detection of markers of inflammation in isolated hearts highlights that SL protects against myocardial injury induced by ischemia via the NF-κB pathway. This result was further verified in H_2_O_2_-induced cells, which indicated that degradation of IκB was a core target for SL.

We successfully established a LAD coronary artery ligation model on anesthetized dogs, and data were gathered from an EECG to calculate the degree and scope of the ST segment. The ST segment increased a degree in the MI group and reduced in the N-ST scope. The myocardial tissue was collected to detect the myocardial infarction size by N-BT staining. The myocardial infarction size was significantly decreased after SL treatment with a 10% decreasing amplitude. As a whole, SL had a protective effect on the ischemic heart and could reduce myocardial infarct size.

In another experiment, the Langendorff-perfused rat heart model was established, and we found that SL also had a same effect on isolated rat hearts by monitoring the parameters of the left ventricular function. As indicators of reflecting and evaluating left ventricular systolic and diastolic function, LVEDP and ± dp/dt_max_, respectively, improved the heart function of the SL treatment after perfusion, which verified the protective effect of SL. In general, SL improved myocardial contractility to ensure well supplied blood to the heart.

Previous pathological studies have shown that IHD and acute myocardial infarction, as main clinical manifestations, have increasingly become a major cause of death (Zhao et al., 2019). IHD and acute myocardial infarctions are associated with atherosclerosis (Esper and Nordaby, 2019), a chronic inflammatory disease that is characterized by the development of cholesterol-rich arterial plaques (Stary et al., 1994) due to several factors, such as hyperlipidaemia, inflammation, oxidative stress, and immune cell infiltration (Viola and Soehnlein, 2015; Libby et al., 2016). Ischemia and necrotic cardiomyocytes would trigger early inflammation through IL-1β signaling and the NF-κB pathway (Latet et al., 2015; Sager et al., 2017; Michels et al., 2019). In the present study, inflammation markers were detected by related kits in isolated hearts, and the results indicated that SL could interfere with myocardial injury under ischemia–reperfusion conditions. SL inhibited the increase in LDH and IL-1β, which may be related to the inflammatory response and lipid peroxidation. Additionally, when cells are stimulated by inflammatory factors, they can produce a large amount of NO. NO is a free radical that can react with a superoxide anion to form a highly oxidized peroxynitrite anion (ONOO^-^), which causes lipid peroxidation and directly damages endothelial cells (Szabó et al., 1996). NO can also increase the expression of adhesion molecules and inflammation factors (Bonafede and Manucha, 2018). In addition, excessive peroxynitrite further leads to increased levels of nitrative tyrosine and decreased biological activity of NO, further causing DNA damage (Nowak et al., 2017). Our study showed that SL had a protective effect against increasing NO levels in myocardial ischemia injury, and had a significant improvement in nitrogen oxidative stress. Similarly, SL treatment groups had significantly decreased levels of eNOS and iNOS compared with the I/R group.

NF-κB is a key factor in the regulation of the inflammatory signaling networks, and the PI3K/Akt signaling pathway may activate NF-κB. For these reasons, our study detected the expression of related inflammatory factors on the PI3K pathway. NF-κB and IL-1β contents were significantly decreased in the SL treatment groups; however, there was no change in PI3K levels. These findings suggested that SL may protect against myocardial ischemia injury by affecting the NF-κB pathway, further regulating the level of the inflammatory response.

Large amounts of ROS, which is one of the main factors of myocardial cells death, can be produced during myocardial ischemia and reperfusion. H_2_O_2_ can simulate the pathological process to establish an ideal model for I/R *in vitro*. Our study used optimized H9c2 cell and stimulated oxidative stress by treating with H_2_O_2_. This H_2_O_2_-induced injury model has been shown in previous studies (Li Y. et al., 2016). H_2_O_2_, a source of ROS, can directly oxidize lipids and proteins on cell membranes. H_2_O_2_ can generate more active free radicals such as ·OH through reacting with iron ions, then induced cell apoptosis or necrosis. After H_2_O_2_-induction, the present study found that LDH, NO, and IL-1β contents had a significant decreased in the SL groups compared with the H_2_O_2_-induced group, which is consistent with the results found in the isolated heart. On this basis, Bio-Plex array analysis was used to further investigate the phosphorylation of related signal molecules and the intervention of SL, which explored eight phosphorylated signaling molecule: p-IκB, p-ERK1/2, p-p38 MAPK, p-Stat3, p-c-JUN, p-IGF-IR, p-MEK, and p-Akt. Twenty-five micrograms per millilitre of SL reduced p-IκB compared with the H_2_O_2_ group, which indicated that p-IκB was a core point for SL to inhibit myocardial ischemic injury.

Our study used the proteasome inhibitor, MG132, and the IKK kinase inhibitor, parthenolide, to observe NF-κB activation pathway. The results showed that MG132 at 5 μM partially reversed the inhibitory effect of SL on the expression of F-actin in H_2_O_2_-induced cells. The effect on NO and IL-1β also decreased; however, parthenolide had no significant effect on the SL-induced response. The results indicated that SL play its role may be mainly related to mechanism modulating degradation of IκB, and affected the activation of NF-κB pathway indirectly.

## Conclusion

In conclusion, SL had a protective effect on myocardial ischemic injury. This conclusion was supported in three ways. First, SL effectively protected the degree and scope of myocardial ischemia and significantly decreased the area of myocardial infarction. SL maintained decent blood supply to the heart by improving myocardial contractility. Second, SL regulated the immune system by effectively reducing IL-1β, NO, iNOS, and eNOS contents. This result suggests modulation of the NF-κB pathway. Finally, SL primarily affected the degradation of IκB by proteasome, as shown through an inhibition study, in an IKK-independent manner. This reduction in IκB degradation caused by SL prevented the nuclear translocation of NF-κB, which affected the activation of the NF-κB pathway. As shown in the myocardial ischemia models of our study, the intervention of inflammatory response may be the core mechanism by which SL played its therapeutic effect. The present results showed that SL could protect against the common pathological cycle between the different stages of atherosclerosis and inflammation. These studies may provide new ideas and tools for intervention of myocardial ischemia cardiovascular disease, and be helpful in the prevention and treatment of cardiovascular diseases in the future.

## Data Availability Statement

All datasets generated for this study are included in the article/**Supplementary Material**.

## Ethics Statement

The animal study was reviewed and approved by Guidance for the Care and Use of Laboratory Animals issued by the Ministry of Science and Technology of China and the Use Committee of Institute of Basic Theory for Chinese Medicine, China Academy of Chinese Medicine Science.

## Author Contributions

YG participated in most of the experiments, analyzed the data and wrote the manuscript. QY contributed to the experiment design and the experiments work. X-GW and Y-JW contributed to the experiment implementation. X-QH performed and analyzed the experiments. X-JZ and Y-JL reviewed the results and approved the version of the manuscript. X-XZ took charge of the research design and coordinated the whole project. All authors contributed to reviewing the results, writing the manuscript, and approving the final version of the manuscript.

## Funding

This work was supported by the National Natural Science Foundation of China (81573649, 81673640), and the experiment was funded by the Institute of Chinese Materia Medica, China Academy of Chinese Medical Sciences.

## Conflict of Interest

The authors declare that the research was conducted in the absence of any commercial or financial relationships that could be construed as a potential conflict of interest.
