# Supplementary material for: Shenlian Extract Against Myocardial Injury Induced by Ischemia Through the Regulation of NF-κB/IκB Signaling Axis
Source: Front Pharmacol. 2020 Mar 6;11:134. doi: 10.3389/fphar.2020.00134 (PMC7069067; doi:10.3389/fphar.2020.00134)
Supplement: Supplementary file 2 [file DataSheet_2.pdf]

Supplementary Experiment of Shenlian Extract Against Myocardial Injury Induced by Ischemia Through the Regulation of NF-κB/IκB Signaling Axis

1 High Performance Liquid Chromatography (HPLC) analysis of Shenlian extract (SL)

Supplementary method and result

SL consisted of *Salvia miltiorrhiza Bunge* extract and *Andrographis paniculata* extract at a ratio of 15:9. Three main components of SL were confirmed by Waters HPLC analysis (Supplementary Figure 1).

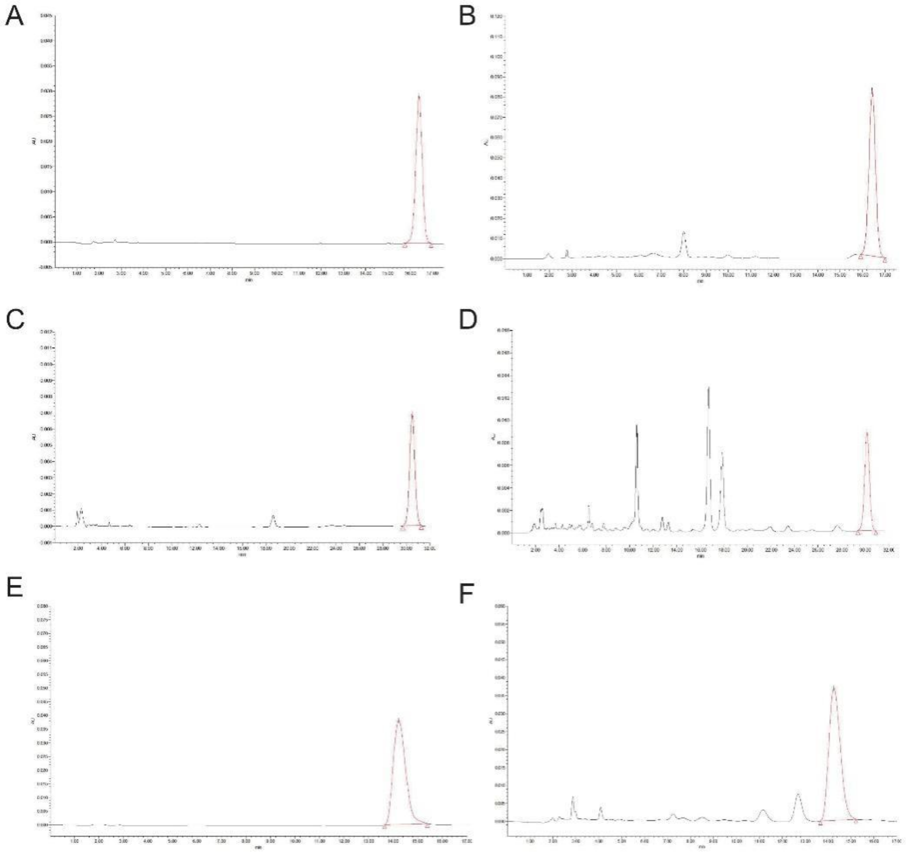

Supplementary Figure 1. Chromatogram of content determination of Shenlian extract (SL) by High Performance Liquid Chromatography (HPLC). (A) HPLC chromatogram for the decision of andrographolide standard substance. (B) HPLC chromatogram for the determination of andrographolide sample. (C) HPLC chromatogram for the determination of tanshinone IIA standard substance. (D) HPLC chromatogram for the resolution of tanshinone IIA sample. (E) HPLC chromatogram for the determination of salvianolic acid B standard substance. (F) HPLC chromatogram for the determination of salvianolic acid B sample.

2 SL protected myocardial ischemia injury in hemodynamic parameters on anesthetized dogs

2.1 Supplementary method of measurement of hemodynamic parameters on anesthetized dogs

To explore the effect of SL on heart function in the myocardial ischemia injury. In the model of myocardial ischemic in anesthetized dogs (same as the myocardial ischemic model in manuscript), a catheter was inserted into the right arteria carotis communis of heart to monitor blood pressure curve (systolic blood pressure and diastolic blood pressure) via a pressure transducer (Ultrasonic doppler flowmeter system, TRITON, USA) connected to MP150 multi-guide physiological record analysis system (BIOPAC, USA) and electromagnetic flowmeter (MF-1100, Japanese photoelectricity). The following parameters of myocardial function were measured after left anterior descending (LAD) coronary artery ligated: the heart rate (HR), the coronary flow (CF), the coronary resistance (CR), the cardiac output (CO), the stroke volume (SV), and the total peripheral resistance (TPR). These data were performed by the rate of the change, it was calculated by: (100%) = each value/ischemia value *100.

2.2 Supplementary result showed that SL protected against myocardial ischemia injury in hemodynamic parameters on anesthetized dogs

Compared with the MI group, there did not show a significant change of HR, CR, and TPR of the SL treatment group (Supplementary Figure 2A, 2C, 2F). CF treatment by SL only showed a significant increase at 45 and 60 min compared with the MI group (Supplementary Figure 2B). Treated by 132 mg/kg of SL, there was significantly increased in CO and SV (Supplementary Figure 2D, 2E), which reflected that SL enhanced the myocardial contractility.

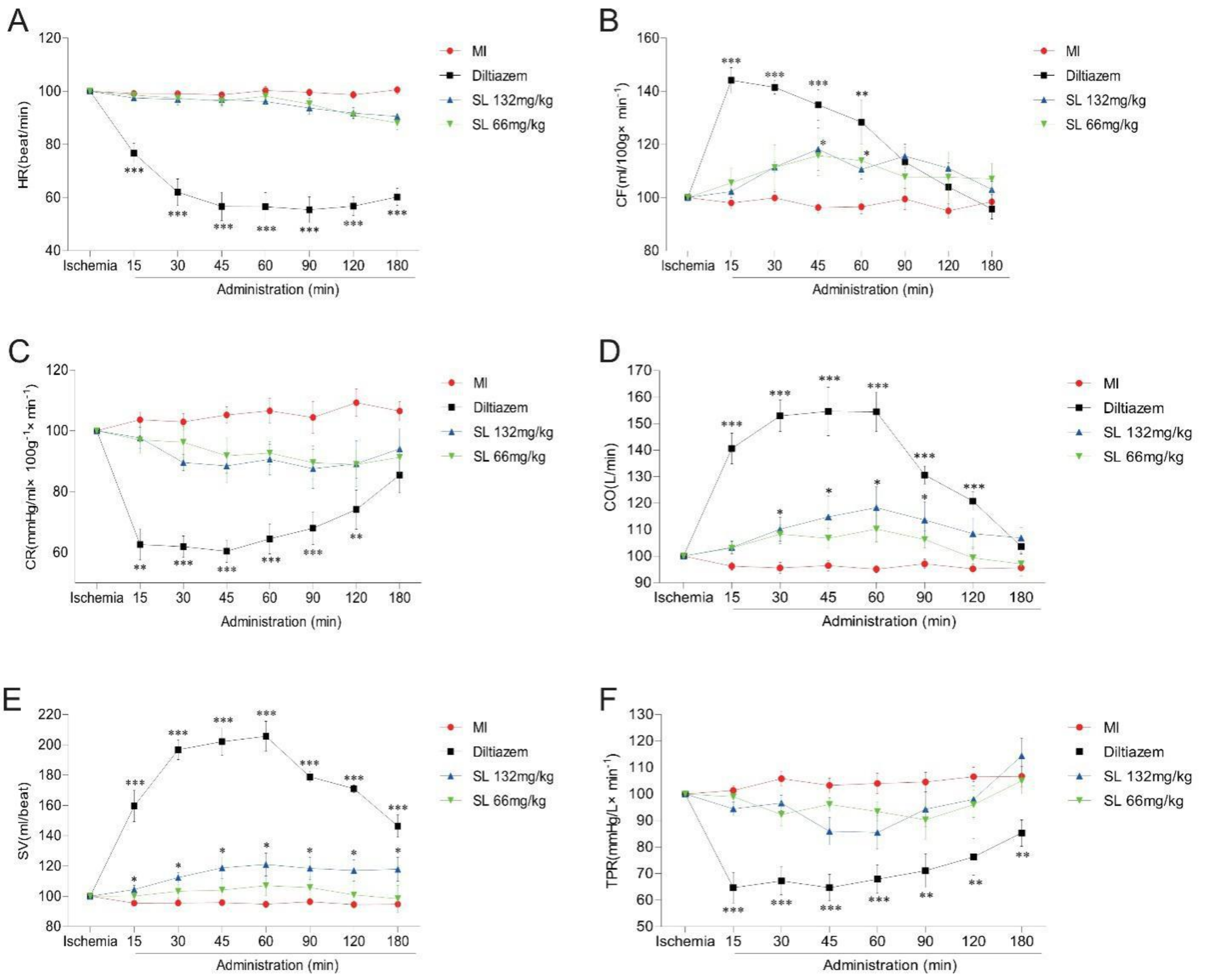

Supplementary Figure 2. Effects of Shenlian extract (SL) on hemodynamic parameters on anesthetized dogs. (A) Changes in heart rate (HR) by myocardial ischemia injury by MP150 detection. (B) Changes in coronary flow (CF) by myocardial ischemia injury calculated by blood pressure curve. (C) Changes in coronary resistance (CR) by myocardial ischemia injury calculated by blood pressure curve. (D) Changes in cardiac output (CO) by myocardial ischemia injury calculated by blood pressure curve. (E) Changes in stroke volume (SV) by myocardial ischemia injury calculated by blood pressure curve. (F) Changes in total peripheral resistance (TPR) by myocardial ischemia injury calculated by blood pressure curve. Results represent mean ± SEM, n=8. Statistical analysis was performed by one-way ANOVA, followed by Dunnett's Multiple Comparison test. * $P < 0.05$, ** $P < 0.01$, *** $P < 0.001$ versus the myocardial ischemia (MI) group.

3 Measurement of cell viability by CCK8 in H9c2 cells

H9c2 cells were conventionally cultured (same as the H9c2 cell culture in manuscript), experiments were divided into 7 groups: an untreated control group (untreated); a H_2O_2-induced group (500 μM

H_2O_2 for 6 h); a 10 μg/mL SL treatment group (SL 10 μg/mL treatment for 24 h, then 500 μM H_2O_2 for 6 h); a 25 μg/mL SL treatment group (SL 25 μg/mL treatment for 24 h, then 500 μM H_2O_2 for 6 h); a 50 μg/mL SL treatment group (SL 50 μg/mL treatment for 24 h, then 500 μM H_2O_2 for 6 h); a 100 μg/mL SL treatment group (SL 100 μg/mL treatment for 24 h, then 500 μM H_2O_2 for 6 h); a 200 μg/mL SL treatment group (SL 200 μg/mL treatment for 24 h, then 500 μM H_2O_2 for 6 h). CCK8 was used to detect cell viability by microplate reader (BioTek ELX800) following the manufacturer's instructions.

The results showed that SL treatment groups had significantly increased the cell viability compared with the H_2O_2-induced group (Supplementary Figure 3).

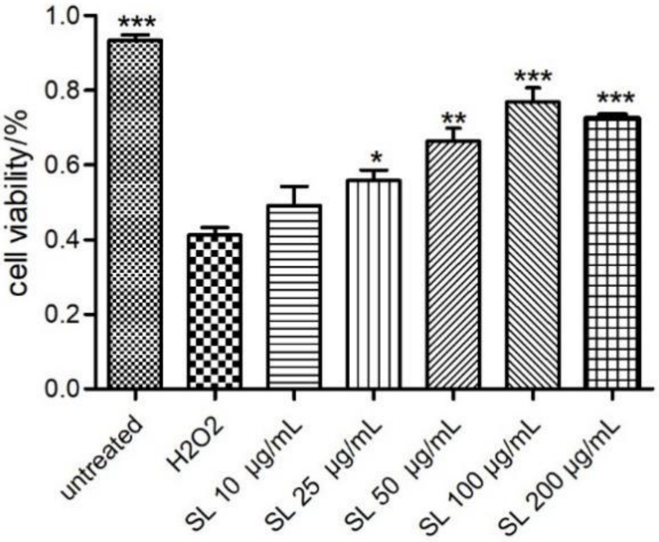

Supplementary Figure 3. The cell viability of Shenlian extract (SL) by H_2O_2 -induced. Results represent mean ± SD, n=6. Statistical analysis was performed by one-way ANOVA, followed by Dunnett's Multiple Comparison Test. * $P < 0.05$, ** $P < 0.01$, *** $P < 0.001$ versus the H_2O_2 -induced (H_2O_2) group.

4 NF-κB was one of the critical pathways for the anti-inflammation effect of SL

4.1 Supplementary method of western blot, immunofluorescence and RT-PCR analysis in NF-κB signaling pathway

To explore the treatment effect with SL inhibiting NF-κB (p65) and PPARγ translocation in TNF-α-activated H9c2 cells. H9c2 cells were divided into 5 groups: an untreated control group (Ctl); a TNF-α-activated model group (TNF-α for 2 h); a 25 μg/mL SL treatment group (SL 25 μg/mL treatment for 24 h, then TNF-α for 2 h); a 50 μg/mL SL treatment group (SL 50 μg/mL treatment for 24 h, then TNF-α for 2 h); a 100 μg/mL SL treatment group (SL 100 μg/mL treatment for 24 h, then TNF-α for 2 h). The homogenate of cells was acquired to measure the expression of NF-κB(p65) and PPARγ by western blotting followed by the manufacturer's instructions strictly.

Furthermore, the NF-κB inhibitor, PDTC (30 and 50 μM), were incubated in culture cells before SL treatment to explore whether protection cells via the NF-κB pathway. The functional pathway was examined by comparing the expression of filamentous actin (F-actin) and inflammatory cytokines in

different groups in H9c2 cells. Staining with fluorescently labelled phalloidin clearly showed the distribution of microfilaments in the cells. The expression of F-actin in the cells was observed by Laser Scanning Confocal Microscope (Olympus, Japan) immediately after labelling, with an excitation wavelength of 488 mn and an emission wavelength of 530 mm, per instructions. The supernatant of cells was acquired to measure the concentrations of IL-1β and IL-6 by RT-PCR, and the detection operations were performed according to the manufacturer's instructions.

4.2 Supplementary result of NF-κB was one of the critical pathways for the anti-inflammation effect of SL

Compared with the control group, the expression of NF-κB p65 significantly increased in the model group, but had no significant change in PPARγ translocation. SL had a markedly inhibiting effect on high expression of NF-κB activated by TNF-α, and little impact on PPARγ (Supplementary Figure 4A). To explore the exact implications of NF-κB, incubated 30 and 50 μM of PDTC in cells. SL with 50 μg/mL decreased expression of F-actin and suppressed of IL-1β and IL-6 activation by TNF-α (Supplementary Figure 4B, 4C, 4D). The 50 μM of PDTC completely reversed the effect of SL. These data suggest that NF-κB was one of the essential pathways for the anti-inflammation effect of SL.

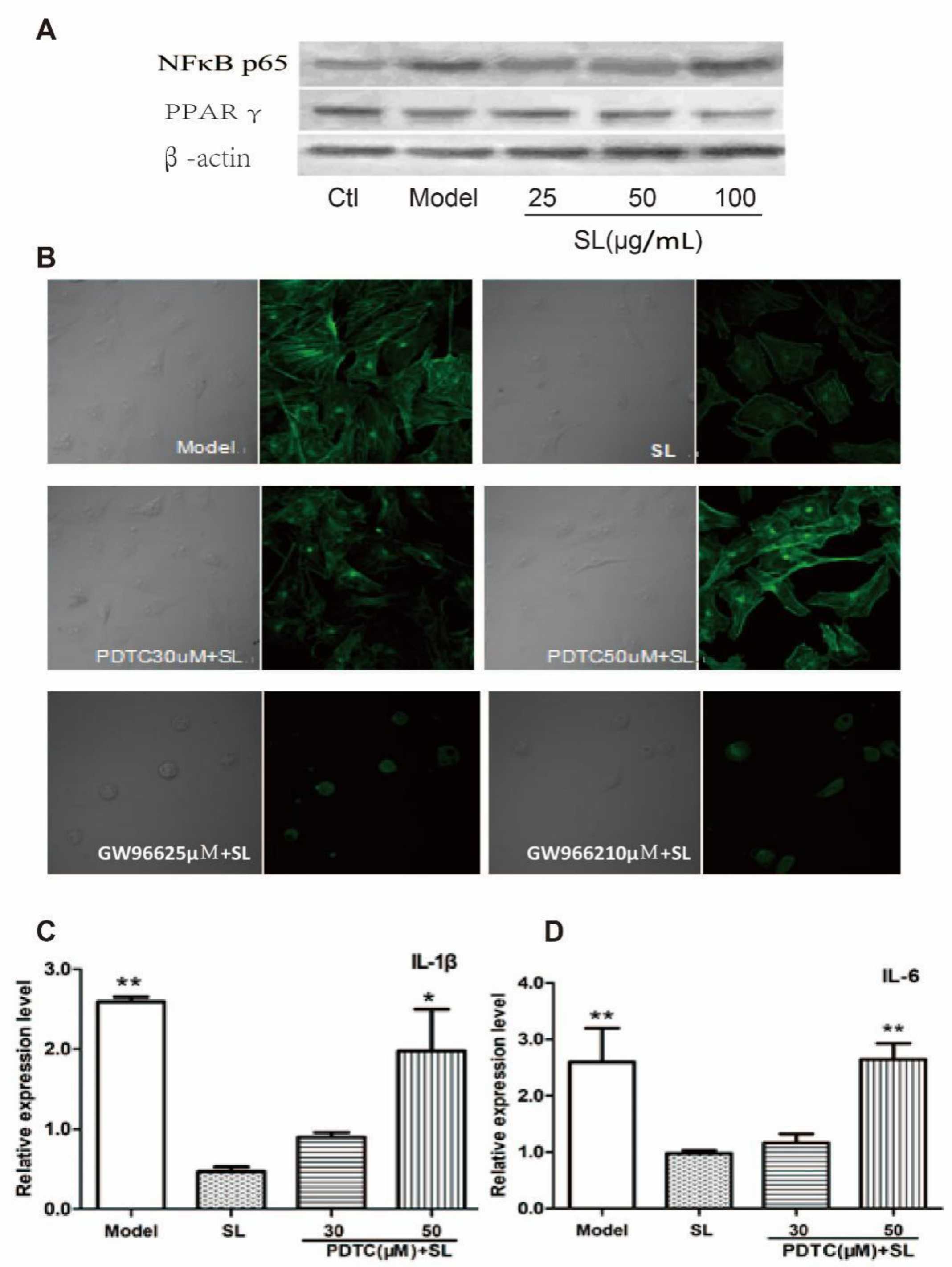

Supplementary Figure 4. Effects of Shenlian extract (SL) on the expression of NF-κB and PPARγ in H9c2 cells. (A) The expression of NF-κB and PPARγ and band intensity. (B) The expression of F-actin after the NF-κB inhibitor (PDTC, 30 and 50μM) and PPARγ inhibitor (GW9662, 5 and 10μM), were added to cultivate cells before SL treatment. Staining with fluorescently labeled phalloidin, which was displayed in green, clearly showed the distribution of F-actin in the cells. Representative image chosen by five visual fields and photos by Laser Scanning Confocal Microscope. (C, D) The expression of IL-1β and IL-6 in H9c2 cells pre-treated with PDTC by RT-PCR. (C, D expressed as

mean ± SD. Statistical analysis was performed by one-way ANOVA, followed by Dunnett's Multiple Comparison Test. *$P < 0.05$, **$P < 0.01$, ***$P < 0.001$ versus the SL treatment group.

5 Supplementary experiment

5.1 Supplementary materials

BMS-345541 (BMS), a highly selective inhibitor of IκB Kinase, was purchased from MedChemExpress LLC (USA). Caffeic Acid Phenethyl Ester (CAPE), a specific inhibitor of activation of nuclear transcription factor NF-κB, was purchased from MedChemExpress LLC (USA).

5.2 Supplementary method of Reverse-transcription PCR (RT-PCR) Assay

In the SL treatment groups, BMS (5 μM) and CAPE (50 μM) were incubated in culture cells with drug treatment for 24 h, respectively (Battula VL et al., 2017; Nie J et al., 2017). The H9c2 cells were divided into 5 groups: a normal control group (NC); a H_2O_2-induced group (500 μM H_2O_2 for 6 h); a 25 μg/mL SL treatment group (SL 25 μg/mL treatment for 24 h, then 500 μM H_2O_2 for 6 h); a 25 μg/mL SL treated with BMS inhibited group (SL 25 μg/mL treatment with 5 μM BMS for 24 h, then 500 μM H_2O_2 for 6 h); a 25 μg/mL SL treated with CAPE inhibited group (SL 25 μg/mL treatment with 50 μM CAPE for 24 h, then 500 μM H_2O_2 for 6 h).

RT-PCR was used to determine the relative RNA expression of Bnip3. Total mRNA was extracted by Trizol reagent (Life Technologies, Grand Island, NY, USA). The protocol of RT-PCR was strictly followed by RevertAid First Strand cDNA Synthesis kit (Thermo Fisher Scientific, Waltham, MA, USA) (Bachman J, 2013). The primers for Bnip3 detection were: Forward 5'-GTGGATATTGTTGCCATCA-3'; Reverse 5'-ACTCATACAGCACCTCAG-3'. The GAPDH primers were: Forward 5'- TCTCAGTGGTCACTTCCCAG-3'; Reverse 5'-TAGTGGAAGTTGTCAGACGCC-3'.

5.3 Supplementary result

NF-κB was one of the important pathways for the protective effect of SL against myocardial ischemic injury

To investigate the exact mechanism of SL treatment, we focused the expression of Bnip3 mRNA were detected by RT-PCR assay. Some studies had shown that NF-κB signaling suppresses transcription of the death gene Bnip3 (Dhingra R et al., 2013). Through analysis of RNA bands and intensity of gray scale value, SL significantly inhibited the expression of Bnip3 compared with model group ($^{##}P < 0.05$, Supplementary Figure 5A, 5B). SL effectively protected against myocardial ischemic injury mainly through NF-κB pathway.

The result of the highly selective inhibitor of IκB Kinase (BMS) and the specific inhibitor of activation of nuclear transcription factor NF-κB (CAPE) was further verified the regulation of NF-κB/IκB signaling axis. When added into 5 μM BMS or 50 μM CAPE, the effect of SL had been completely reversed (Supplementary Figure 5A, 5B), indicating that SL protected against myocardial ischemic injury by regulate of NF-κB/IκB signaling axis.

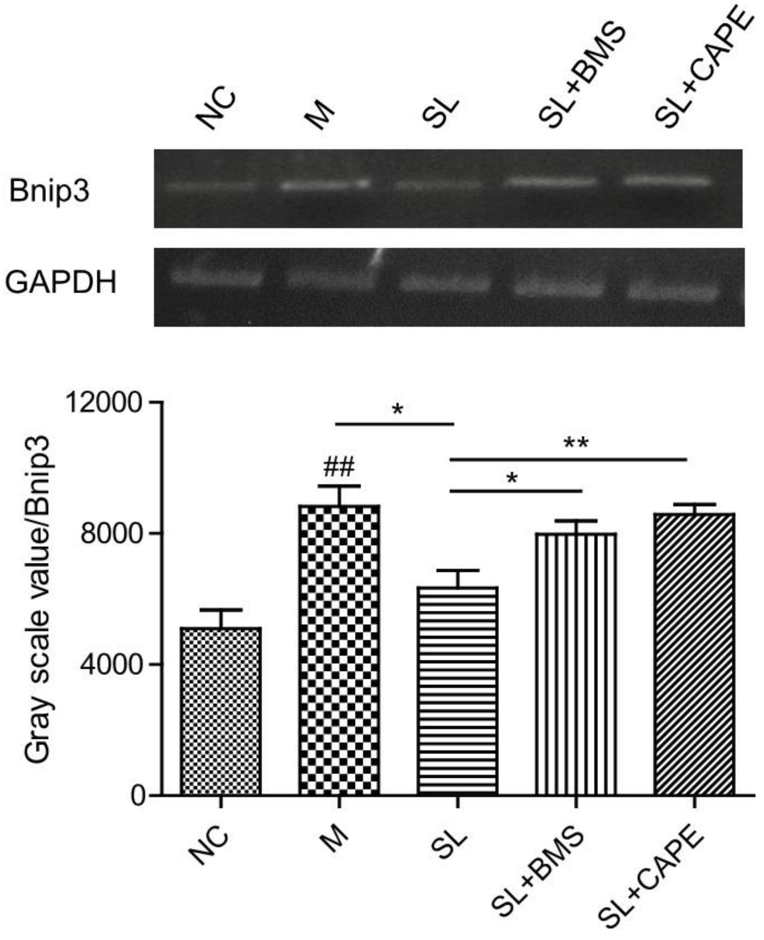

Supplementary Figure 5. Effects of Shenlian extract (SL) on the expression of Bnip3 in H9c2 cells. (A) The expression of Bnip3 band intensity by RT-PCR. (B) The gray scale value of Bnip3 band intensity calculated by Image J. Detected the effect on expression of Bnip3 by SL treatment and the effect of inhibtors (BMS and CAPE) on SL treatment synchronously. B expressed as mean ± SEM of five independent experiments. Statistical analysis was performed by one-way ANOVA, followed by Dunnett's Multiple Comparison Test. $^{\#\#}P < 0.01$ versus the NC group, $^{*}P < 0.05$， $^{**}P < 0.01$ versus the SL group.

References

Battula, V. L., Nguyen, K., Sun, J., Pitner, M. K., Yuan, B., Bartholomeusz, C., et al. (2017). IKK inhibition by BMS-345541 suppresses breast tumorigenesis and metastases by targeting GD2+ cancer stem cells. *Oncotarget.* 8(23): 36936-36949. doi: 10.18632/oncotarget.16294

Nie, J., Chang, Y., Li, Y., Zhou, Y., Qin, J., Sun, Z., et al. (2017). Caffeic Acid Phenethyl Ester (Propolis Extract) Ameliorates Insulin Resistance by Inhibiting JNK and NF-κB Inflammatory Pathways in Diabetic Mice and HepG2 Cell Models. *J Agric Food Chem.* 65(41):9041-9053. doi: 10.1021/acs.jafc.7b02880

Bachman, J. (2013). Reverse-transcription PCR (RT-PCR). *Methods Enzymol.* 530:67-74. doi: 10.1016/B978-0-12-420037-1.00002-6

Dhingra, R., Gang, H., Wang, Y., Biala, A. K., Aviv, Y., Margulets, V., et al. (2013). Bidirectional regulation of nuclear factor-κB and mammalian target of rapamycin signaling functionally links

Bnip3 gene repression and cell survival of ventricular myocytes. *Circ Heart Fail.* 6(2):335-43. doi: 10.1161/CIRCHEARTFAILURE.112.000061
